# Case Report: Successful ABO-Incompatible Deceased Donor Kidney Transplantation in an Infant Without Pre-transplant Immunological Treatment

**DOI:** 10.3389/fmed.2022.838738

**Published:** 2022-03-02

**Authors:** Daqiang Zhao, Lan Zhu, Shengyuan Zhang, Zhiliang Guo, Lu Wang, Tianhui Pan, Rula Sa, Zhishui Chen, Jipin Jiang, Gang Chen

**Affiliations:** ^1^Institution of Organ Transplantation, Tongji Hospital, Tongji Medical College, Huazhong University of Science and Technology, Wuhan, China; ^2^Key Laboratory of Organ Transplantation, Ministry of Education, Ministry of Public Health, Chinese Academy of Medical Sciences, Wuhan, China

**Keywords:** ABO incompatibility, infant, kidney transplantation, deceased donor, pediatric transplantation

## Abstract

ABO blood group antibodies have not been generated or are at low titer during early infancy. Therefore, in theory, ABO-incompatible kidney transplantation (ABOi KT) may be successfully achieved in small infants without any pre-transplant treatment. We report here the first ABO-incompatible deceased donor kidney transplantation (ABOi DDKT) in an infant. The recipient infant was ABO blood group O, and the donor group A. The recipient was diagnosed with a Wilms tumor gene 1 (WT1) mutation and had received peritoneal dialysis for 4 months prior to transplant. At 7 months and 27 days of age, the infant underwent bilateral native nephrectomy and single-kidney transplantation from a 3-year-old brain-dead donor. No pre- or post-transplantation antibody removal treatment was performed, since the recipient's anti-iso-hemagglutinin-A Ig-M/G antibody titers were both low (1:2) before transplantation and have remained at low levels or undetectable to date. At 11 months post-transplant, the recipient is at home, thriving, with normal development and graft function. This outcome suggests that ABOi DDKT without antibody removal preparatory treatment is feasible in small infants, providing a new option for kidney transplantation in this age range.

## Introduction

Blood group ABO-incompatible kidney transplantation (ABOi KT) has been an effective strategy to relieve donor kidney shortage and reduce waitlist mortality of patients with end-stage renal disease (1). Appropriate immunological preparations to remove blood group antibody or suppress its generation, such as administration of rituximab, plasmapheresis, immunoadsorption, inhibition of complement activation, and powerful maintenance immunosuppression, are usually a prerequisite for successful ABOi KT (2–5). Because of time constrains associated ABO-incompatible deceased donor kidney transplantation (ABOi DDKT) (the allocated deceased donor organ needs to be transplanted within a very short time), ABOi KT was originally piloted and is mostly performed in living-donor kidney transplantation (ABOi LDKT) (1).

Blood group ABO antibodies in the serum are formed naturally. Their production is stimulated when the immune system encounters the “missing” ABO blood group antigens in foods or micro-organisms (6). Iso-hemagglutinins are not present on red blood cells in new born infants, and serum blood group antibody anti-A and anti-B titers increase gradually from 3 to 12 months but usually remain low until 12–14 months of life (7). Also, the complement system is not fully competent in young infants (8). Thus, the primary risk factors for hyperacute rejection mediated by blood group antibodies are absent during early infancy. ABOi deceased donor heart transplantation has been reported to be clinically achievable in infants with long-term success (9, 10). However, no successful cases of ABOi DDKT have been reported in infants to date.

We reasoned that ABOi DDKT would be safe during early infancy because of the recipients' relative immunologic immaturity. We therefore performed ABOi DDKT in an infant without any preparatory blood group antibody-removal procedures either pre- or post-transplantation. To our knowledge, this is the first report of ABOi DDKT in an infant.

## Case Report

The kidney transplant recipient, a male infant, was born at 38 weeks gestational age with a birth weight of 3,370 g. He presented with disturbed sleep and had recurrent diarrhea and abdominal distension since birth. Various oral probiotic supplements were administered, and he was treated by doctors of traditional Chinese medicine but did not improve. The patient developed nausea and vomiting at about 2 months after birth, concomitant with fever and eclampsia. After symptomatic treatment, the fever and eclampsia improved, but symptoms of vomiting, diarrhea, and abdominal distension worsened. At the age of 3 months, he developed apparent oliguria. Subsequent clinical laboratory tests indicated a significant increase in serum creatinine (>500 μmol/L). The infant was then admitted to the pediatric intensive care unit (PICU) for therapy and was diagnosed with kidney dysfunction with end-stage renal disease, chronic diarrhea, severe anemia, decompensated metabolic acidosis, dilutional hyponatremia, pleural effusion, and hypertension. Further genetic testing confirmed the diagnosis of a Wilms tumor gene 1 (WT1) exon 9 heterozygous mutation.

The recipient then received regular continuous renal replacement treatment (CRRT) through vascular access via catheterization of the right femoral vein. His hemoglobin level dropped to 35 g/L and his platelet was count 10 × 10^9^/L. Infusions of red blood cells and thrombocytes were performed as symptomatic therapy. After 16 days of PICU hospitalization and CRRT, the patient was transferred to a general ward for follow-up treatment. During his time in the PICU, he developed catheter-related sepsis and bilateral bronchiolitis. Optimized anti-biotics were used, and catheter replacement and a ventilator-assisted breathing were performed to treat these complications. Ten days after the infant was moved out of the PICU, he received peritoneal dialysis catheterization and laparoscopic orchiopexy. The infant recovered uneventfully from these surgical procedures and then was gradually switched from CRRT to regular peritoneal dialysis. At the age of 3 months, he was referred to our institution for kidney transplantation.

**Figure 1:**
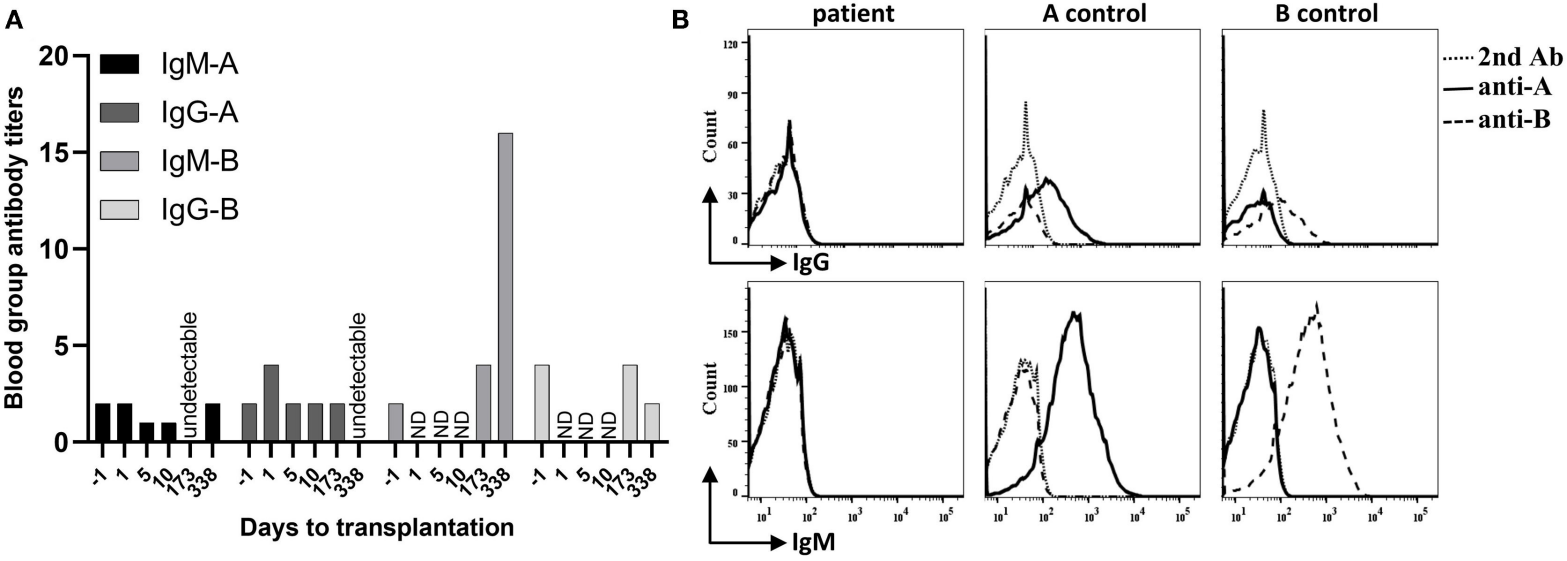
Recipient blood group antibody titers and hemagglutinin expression on red blood cells (RBC). **(A)** The recipient infant ABO blood group is O, and the donor group A. Within post-operative day 338, recipient anti-donor iso-hemagglutinin-A IgM (IgM-A) titer remained at low levels (equal or less than pre-transplant level 1:2) or declined to undetectable level. Recipient anti-donor iso-hemagglutinin-A IgG (IgG-A) titer increased on post-transplantation day 1, then stabilized at the pre-transplant level (1:2) until day 173 and declined to undetectable level on day 338. In contrast, recipient's IgM-B titer significantly increased (1:16 on day 338), and the IgG-B titer remained stable for 6 months after transplantation. ND, not done. **(B)** The recipient blood sample was harvested on day 338 after transplantation. After incubation with anti-A or anti-B hemagglutinin antibodies and wash, samples were incubated with fluorescein isothiocyanate (FITC) or Phycoerythrin (PE) conjugated second antibodies (2nd Ab). Cell counts were gated on CD45- whole blood cells by flow. The blood group O recipient RBCs did not express either A or B hemagglutinin. The control blood type A and B groups expressed A and B hemagglutinins on their RBCs, respectively.

In the ensuing months of dialysis and waitlisting for a blood group O-matched pediatric donor kidney, the infant developed recurrent pneumonia and peritonitis. He was admitted to the PICU 3 times because of life-threatening illness. After 4 months of waitlisting, a kidney from a 3-year-old donor donated after brain death from cerebral trauma became available in our hospital. However, the donor was ABO-incompatible. The recipient infant was ABO blood group O, and the donor group A. Pre-transplant measurements using reported microcolumn gel card method (11) showed the recipient anti-donor iso-hemagglutinin-A IgM and IgG titers to be low, at 1:2 (Figure 1A). Because of the complications of waiting, the decision was made to perform ABO-incompatible single-kidney transplantation (1 HLA-A match, HLA-B/DR/DQ mismatch), with parental consent and approval by the ethics committee.

**Figure 2:**
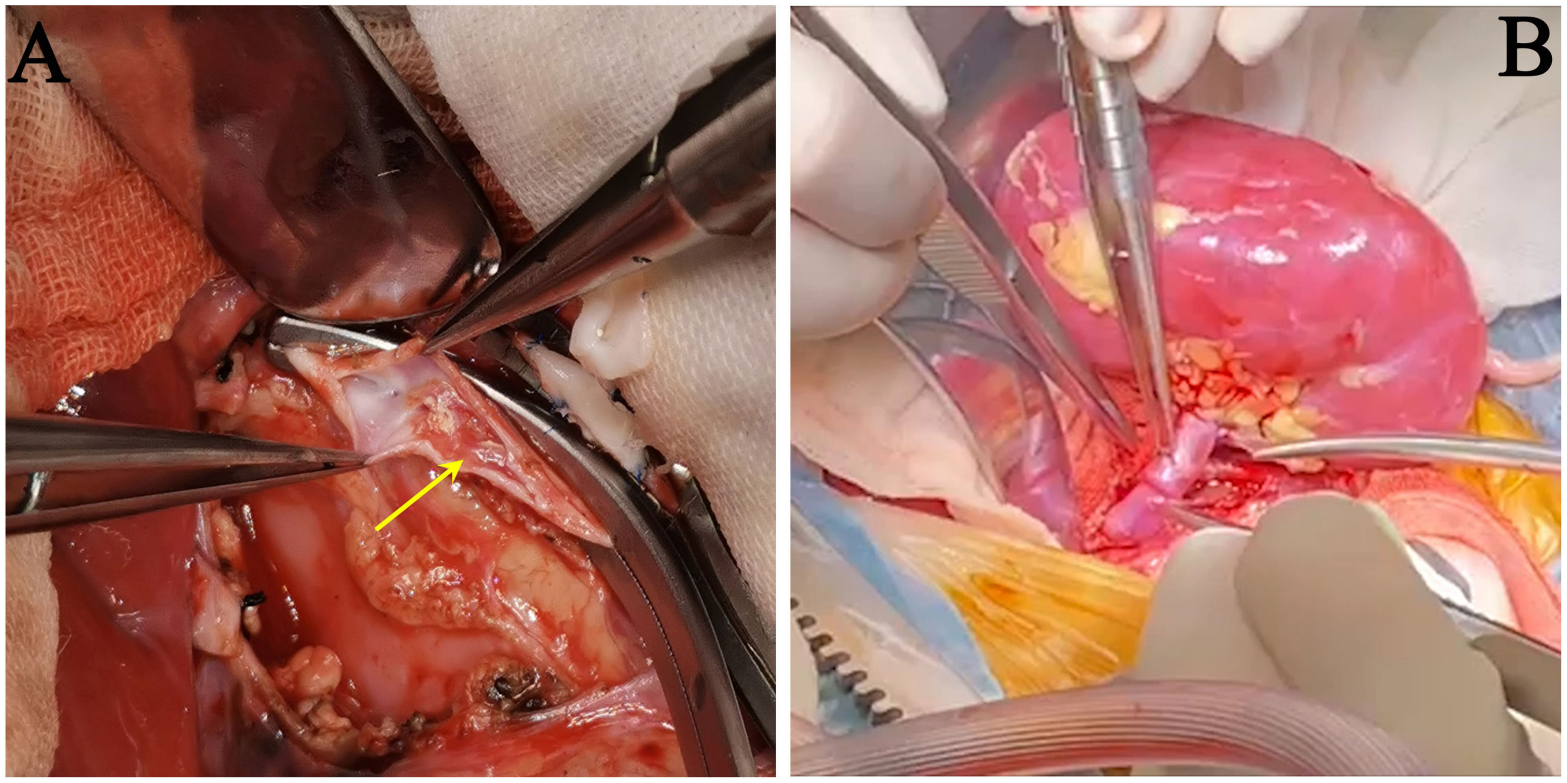
Images during surgery. **(A)** Removal of the thrombo-embolus inside the recipient's inferior vena cava, caused by catheterization from the right femoral vein (yellow arrow). **(B)** The renal artery and vein were end-to-side anastomosed onto the recipient's abdominal aorta and inferior vena cava. The kidney graft was perfectly perfused after the Satinsky vascular clamps were released.

Through a midline abdominal incision, the bilateral native kidneys with WT1-related renal disease were removed, and the new renal allograft was placed in the right flank inside the abdomen. With the aid of surgical loupes (2.5× magnification), a large donor aortic patch (with ostia of renal artery on it) was anastomosed end-to-side to the recipient's abdominal aorta using a running 7-0 Prolene suture. Before the arterial anastomosis was closed, the artery was flushed with a sodium chloride/papaverine solution (3 mg/ml) to prevent renal vasospasm after blood reperfusion. The donor's renal vein with a Carrel vein cuff was anastomosed end-to-side to the recipient's inferior vena cava, using a running 7-0 Prolene suture. During the transplant surgery, the catheter placed through right femoral vein had to be retracted, since the cranial catheter tip end reached the vena cava and caused mural thrombosis inside the vena cava. We had to clear the thrombo-embolus to make an idea site for venous anastomosis (Figure 2A). The ureter was sutured to the recipient bladder using the Lich-Gregoir extravesical technique (12). A 5-F double-J stent was placed temporarily into the ureter. All surgical procedures were uneventful. The graft was perfectly perfused after the Satinsky vascular clamps were released (Figure 2B).

**Figure 3:**
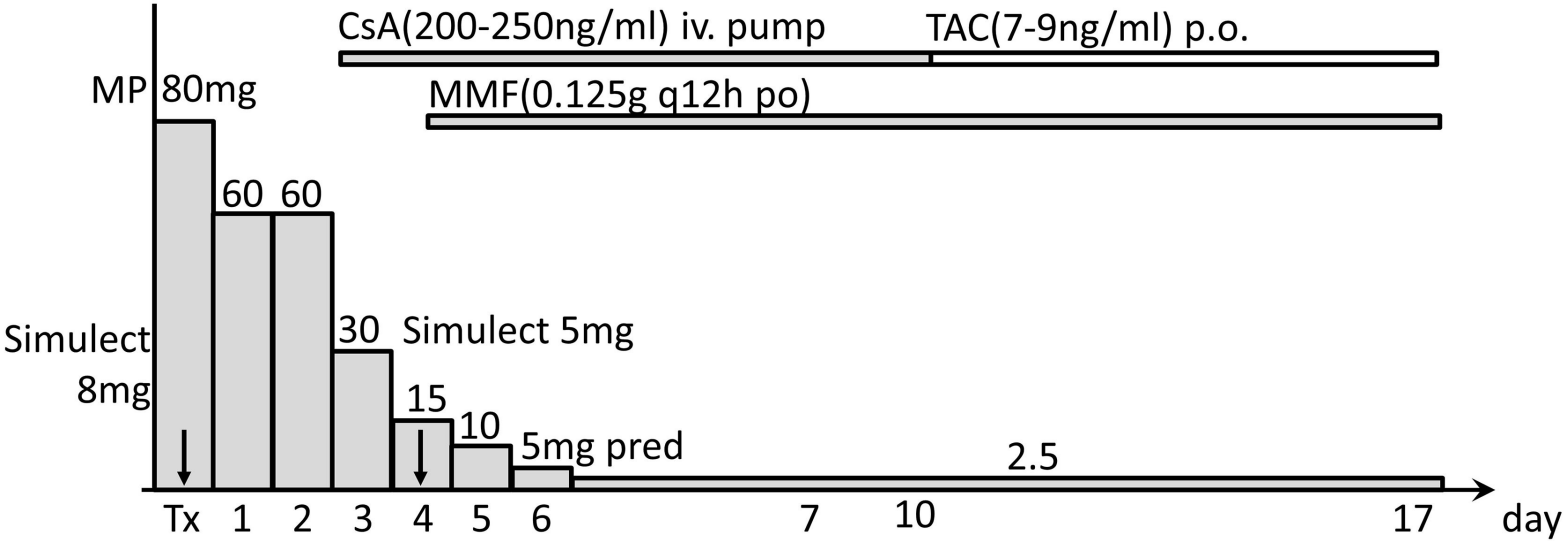
Immunosuppressive regimen used after transplantation. Post-operative immunosuppression included Simulect basiliximab (induction), cyclosporine (CsA), tacrolimus (TAC), mycophenolate mofetil (MMF), methylprednisolone (MP), and prednisone (pred). Tx, transplantation day 0; arrows indicate days of using Simulect.

The infant did not receive any procedures for blood group antibody removal either before or after transplantation. Post-operative immunosuppression included basiliximab (induction), cyclosporine (first), tacrolimus (later), mycophenolate mofetil, methylprednisolone and prednisone (Figure 3). The immunosuppression regimen and perioperative management were based on our pediatric kidney transplantation protocol (Simulect, iv, 20 mg (BW > 35 kg), otherwise 10 mg, d0 and d4; methylprednisolone, iv, 10 mg/kg d0 and d1, 5 mg/kg d2, 2.5 mg/kg [minimum 20 mg] d3, 10 mg d4–5; prednisone, po, 1.2 mg/kg [can be substituted with methylprednisolone tablets (Medrol)] d6; then prednisone tapered by 10 mg every other day to 0.2 mg/kg for maintenance; the cyclosporine pump started on d2 with 2 mg/kg/d and a target trough level at 200–250 ng/ml for recipients younger than 2 years old; the cyclosporine replaced with tacrolimus [target trough level: 7–9 ng/ml] when patient normally resumes oral intake).

**Figure 4:**
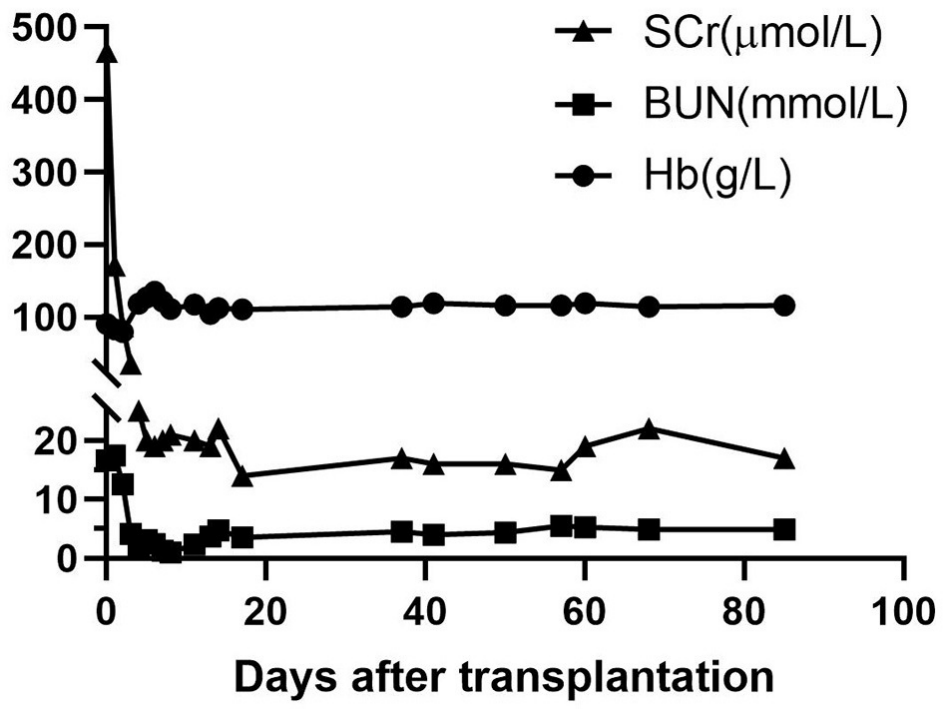
Graft function and hemoglobin levels after transplantation. After transplantation, graft function recovered uneventfully, with serum creatinine (SCr) and blood urea nitrogen (BUN) rapidly declining to normal levels within 10 days post-transplantation. Recipient hemoglobin (Hb) recovered and stayed stable after transplantation.

In the early period after the transplantation, the infant received CRRT twice to treat severe acidosis, possibly caused by the cumulative side effects of consecutively administering sodium nitroprusside to lower his blood pressure. He recovered smoothly otherwise. Graft function was uneventfully recovered, with serum creatinine and blood urea nitrogen rapidly declining to normal levels within 10 days after transplantation (Figure 4). Measurement of blood group antibodies following transplantation showed that the anti-donor iso-hemagglutinin-A IgM titer remained at low levels (equal or less than pre-transplant level 1:2) or declined to an undetectable level and IgG increased to 1:4 on post-transplantation day 1, then stabilized at the pre-transplant level (1:2) until day 173 and declined to undetectable on day 338. However, the anti-non-donor blood group antigen B IgM titer significantly increased to 1:16 on day 338 and IgG did not decline until day 173 (1:4) but declined on day 338 (<1:2) after surgery (Figure 1A). Measurements of the expression of ABO hemagglutinins by flow showed neither A nor B hemagglutinin was present on the recipient red blood cell (RBC) on day 338 after transplantation (Figure 1B).

At 11 months post-transplantation, the infant is thriving (height: 25th percentile, weight: 20th percentile) at home, is developmentally appropriate, and has normal graft function (last serum creatinine level: 33 μmol/L).

## Discussion

The WT1 gene mutations observed in patients with Denys-Drash and Frasier syndrome are associated with genitourinary development and tumorigenesis. Kidney transplantation is recommended for children who progress to real failure (13). Our infant with a WT1 mutation had presented with a series of symptoms of illness since birth and had progressed to renal failure at 3 months of age. The indication for kidney transplantation in this infant was clear and rational. Since severe complications can occur repeatedly while an infant is waiting for renal transplantation, as they did in our case, an earlier renal transplantation can save the child's life. Because in our institution, the baby's blood group was type O, we were unlikely to find a type O donor kidney of a suitable size in a short period of time. The ability to perform successful ABOi KT in such infants will help to shorten the waiting time for transplantation.

The developmental patterns of ABO iso-hemagglutinins is correlated with age (7). The relatively low anti-A and anti-B blood group antibody titers during infancy offers the possibility of performing ABO-incompatible organ transplantation with a simplified pre-treatment, or even without any pre-treatment. On the basis of this theory, the ABOi deceased donor heart (9, 10, 14), liver (15, 16), and lung (17) transplantations have already been successfully performed in infants. Most of these recipient infants received only perioperative plasma exchange, followed by standard immunosuppression without using B-cell depletion reagent rituximab. Dynamically monitoring the generation of blood group antibody after transplantation in these infants indicated that the anti-donor iso-hemagglutinins antibodies are maintained at low levels (9, 10, 14–17). Investigations into the underlying mechanisms involved have suggested that a donor-specific B-cell tolerance to blood group hemagglutinins is established after ABOi deceased donor heart transplantation in infants (18, 19).

Despite of the successful implementation of such ABO-incompatible deceased organ transplantation in infants, until now, ABOi DDKT had not been reported in infants. Kidney allografts seem to be more tolerogenic than are heart allografts (20). ABOi LDKT has been successfully performed in pediatric recipients with relatively low levels of blood group antibodies by using rituximab without anti-A/B antibody removed (21, 22). We therefore reasoned that performing ABOi DDKT is theoretically safe and feasible in small infants who have even lower levels of blood group antibodies. In our case, the infant recipient's anti-A and anti-B titers of IgM/G before transplantation both were 1:2, which are acceptable levels according to ABOi KT protocols (2). Intriguingly, measurements following transplantation demonstrated the anti-donor blood group A IgM titer became undetectable or remained at pre-transplant low level, and the IgG titer first increased to 1:4 (possibly related to blood infusion) but quickly fell to 1:2 within 6 months then undetectable level on day 338 after transplantation. However, the non-anti-donor blood group antibody titer of anti-B IgM at 6 months post-transplantation increased (1:4) and was significantly higher on day 338 (1:16) than the pre-transplant level (1:2). This immunological phenomenon suggests that a specific tolerance to donor-derived blood group antigen iso-hemagglutinin-A may have been generated after ABOi DDKT in our case. This tolerance was not induced by the recipient hemagglutinin-A expression since they were not present on the recipient red blood cells (Figure 1B). This outcome was consistent with ABOi deceased donor heart transplantation cases in infants that have been reported (9, 23). The decline of anti-B IgG titer on day 338 after transplantation (<1:2) was probably associated with oral immunosuppression.

In summary, we report the first successful ABOi DDKT performed in an infant without any immunological pre-treatment. This outcome suggests that ABOi DDKT is feasible in small infants whose ABO blood group antibodies have not yet appeared or are at low titer, providing another option to offer life-supporting kidney transplantation to this age group, especially, the cases with severe disease and rare opportunities to receive a blood type matched donor kidney.

## Data Availability Statement

The raw data supporting the conclusions of this article will be made available by the authors, without undue reservation.

## Ethics Statement

The studies involving human participants were reviewed and approved by Medical Ethics Committee, Tongji Hospital, Tongji Medical College, Huazhong University of Science and Technology. Written informed consent to participate in this study was provided by the participants' legal guardian/next of kin. Written informed consent was obtained from the individual(s), and minor(s)' legal guardian/next of kin, for the publication of any potentially identifiable images or data included in this article.

## Author Contributions

GC, LZ, DZ, and ZG performed the surgery. DZ wrote the manuscript. LZ and GC revised and edited the manuscript. LW performed the flow. All authors participated in patient management and data collection. All authors contributed to the article and approved the submitted version.

## Funding

This study was supported by Tongji Hospital Clinical Research Flagship Program (2019CR108) and National Natural Science Foundation of China (82170774).

## Conflict of Interest

The authors declare that the research was conducted in the absence of any commercial or financial relationships that could be construed as a potential conflict of interest.

## Publisher's Note

All claims expressed in this article are solely those of the authors and do not necessarily represent those of their affiliated organizations, or those of the publisher, the editors and the reviewers. Any product that may be evaluated in this article, or claim that may be made by its manufacturer, is not guaranteed or endorsed by the publisher.

## Abbreviations

WT1: Wilms tumor gene1
ABOi KT: ABO-incompatible kidney transplantation
ABOi LDKT: ABO-incompatible living donor kidney transplantation
ABOi DDKT: ABO-incompatible deceased donor kidney transplantation
PICU: pediatric intensive care unit
CRRT: continuous renal replacement treatment.
